# A review of cervical spine MRI in ALS patients

**Published:** 2018

**Authors:** F Antonescu, M Adam, C Popa, S Tuţă

**Affiliations:** * National Institute of Neurology and Neurovascular Diseases, Bucharest; ** “Carol Davila” University of Medicine and Pharmacy, Bucharest; *** MEDINST Diagnostic Center, Bucharest

**Keywords:** motor neuron disease, amyotrophic lateral sclerosis, multiparametric MRI, spinal cord atrophy, diffusion tensor imaging

## Abstract

**Rationale.** In recent years, significant advances have been made on the subject of MRI examination techniques, which have opened new avenues of research regarding the spinal involvement in amyotrophic lateral sclerosis (ALS).

**Objective.** Our objective was to compile and analyze the available literature data, concerning the MRI of the cervical spine in ALS, detailing the metrics and their significance in diagnosis and follow-up.

**Methods and results.** We have conducted an extensive search on the subject using literature data published over the last fifteen years, correlating it with our own experience.

In ALS, there is a permanent interest in developing new biomarkers that might be sensitive to spatial and temporal patterns of neurodegeneration, which will permit early diagnosis and hopefully lead to new therapeutic approaches. Both diffusion tensor imaging (DTI) and spinal cord morphometry (especially spinal atrophy) reflect different aspects of the disease and correlate with clinical deterioration. Newer approaches like inhomogeneous magnetization transfer (ihMTR) and multiparametric analysis seem to have better sensitivity, are more appropriate for follow-up, and lend themselves to prognostic conclusions.

**Discussion.** We conclude that MRI is a constantly expanding field, a unique non-invasive tool with immense potential in evaluating the in vivo evolution of the neurodegenerative ALS process, both structurally and functionally, with high hopes for the future.

**Abbreviations:** ALS - amyotrophic lateral sclerosis, UMN - upper motor neuron, LMN - lower motor neuron, EMG – electromyography, CST - cortico-spinal tract, FLAIR – fluid-attenuated inversion recovery, MND - motor neuron disease, DTI - Diffusion tensor imaging, FA - fractional anisotropy, MD - mean diffusivity, ihMTR - inhomogeneous magnetization transfer, fMRI - functional MRI

## Introduction

Amyotrophic lateral sclerosis (ALS) is a progressive disease characterized by the combined degeneration of lower and upper motor neurons (LMN and UMN, respectively) leading inexorably to severe neurological deficits and death.

Despite extensive ongoing research, the diagnosis remains a clinical one, with substantial support from electrophysiological studies, mainly electromyography (EMG) (**1**)(**2**). The diagnosis of ALS is especially challenging during the early stages, and the cases where a patient has to be reevaluated at 6-12 months for a clear verdict are not rare. The diagnostic delay is on average one year from the first clinical signs, which leads to the fact that most patients are included in trials after their disease has already advanced significantly (**3**). The current Awaji diagnostic criteria (**Table 1**) have a sensitivity of only 70-80% and, although they have proven superior to the revised El Escorial Criteria, there is a constant desire to improve them (**4**). Cervical spine MRI is currently used for satisfying criteria *1B2***—**i.e., to exclude “other disease processes that might explain the observed clinical and electrophysiological signs”. While this aspect will never fade, the current aspiration is to extend its use to criteria *1A2* and *1A3*, in order to be able to use MRI to gather “evidence of UMN degeneration“ (perhaps also LMN degeneration) and “progressive spread of signs within a region or to other regions”, respectively.

**Table 1:** Awaji-shima consensus recommendations for the application of electrophysiological tests to the diagnosis of ALS, as applied to the revised El Escorial Criteria (1)

**1. PRINCIPLES** (from the Airlie House criteria) The diagnosis of ALS requires (A) the presence of 1. evidence of LMN degeneration by clinical, electrophysiological or neuropathological examination 2. evidence of UMN degeneration by clinical examination; and 3. progressive spread of symptoms or signs within a region or to other regions, as determined by history, physical examination or electrophysiological tests (B) the absence of 1. electrophysiological or pathological evidence of other disease processes that might explain the signs of LMN and/or UMN degeneration, and 2. neuroimaging evidence of other disease processes that might explain the observed clinical and electrophysiological signs
**2. DIAGNOSTIC CATEGORIES** *Clinically definite ALS* is defined by clinical or electrophysiological evidence by the presence of LMN as well as UMN signs in the bulbar region and at least two spinal regions or the presence of LMN and UMN signs in three spinal regions *Clinically probable ALS* is defined on clinical or electrophysiological evidence by LMN and UMN signs in at least two regions with some UMN signs necessarily rostral to the LMN signs *Clinically possible ALS* is defined when clinical or electrophysiological signs of UMN and LMN dysfunction are found in only one region; or UMN signs are found alone in two or more regions; or LMN signs are found rostral to UMN signs. Neuroimaging and clinical laboratory studies will have been performed and other diagnoses must have been excluded

There is increasing interest in developing biomarkers that might be sensitive to spatial and temporal patterns of neurodegeneration, capable to discriminate between different clinical forms and prognostic categories (**5**)(**6**). There is hope that by studying patients in the early clinical stages, perhaps even in the preclinical phase, we could get new insights into ALS pathogenesis and, hopefully, new therapeutic approaches.

While LMNs are relatively easy to assess both clinically and through EMG, UMNs are clinically limited to the modification of reflexes and a positive Babinski sign, both having low specificity and both at risk of being concealed through advanced LMN deterioration. Also, the paraclinical means of assessing UMN at the spinal level are scarce, consisting mainly of transcranial magnetic stimulation. In the last ten years, magnetic resonance imaging (MRI) has been progressing in this direction, the expectations being that it will soon become the main biomarker for corticospinal tracts degeneration. (**7**–**9**).

In comparison to brain imaging, spinal cord MRI has the advantage of simultaneously investigating the upper and lower motor neurons.

In this review, we shall focus on the different aspects of the spinal MRI examination, identifying frequently encountered alterations and their significance for the daily neurological practice, as well as potential surrogate diagnostic markers, stratification and monitoring disease progression of ALS for the future trials.

## The standard protocols

*T1-weighted native images* are the method of choice for the morphometry of anatomical structures. The parameters commonly studied are the thickness or diameter, surface and volume of different structures (**10**).

ALS is known to be associated with signal anomalies of the CST on both brain and spinal MRI, usually in the form of hyperintensity on *T2-weighted images* and fluid-attenuated inversion recovery (FLAIR) sequences. The changes are generally more pronounced in the case of advanced disease (**11**).

T2/FLAIR hyperintensity of the CST is a usual finding in ALS at all levels, but especially in the posterior limb of the internal capsule, in some studies being identified in up to 96% of patients (**12**). The sensitivity and specificity of these changes are very low and have failed to become significant in the diagnosis or patient follow-up (**13**). The same study that found anomalies in 96% of ALS patients, found anomalies in 68% of controls (**12**)(**14**).

ALS patients with CST hyperintensity and prominent UNM involvement are significantly younger, have faster progression rate and shorter survival period compared to those without CST hyperintensity. The reason and the underlying mechanism are unknown (**15**).

The standard protocols remain useful for the exclusion of other pathologies that could mimic motor neuron disease (MND) such as external compression of the spinal cord, syringomyelia, myelitis, intramedullary tumors.

## Diffusion tensor imaging (DTI)

*Diffusion-weighted imaging* (DWI) is an MRI technique which acquires information about the Brownian translation motion of water molecules in tissues. D*iffusion tensor imaging* (DTI) is derived from the DWI data and calculates tensors that can be used to describe fiber bundles in the white matter and produce tractography images. The diffusion tensor is characterized through measures such as fractional anisotropy (FA), which describes how strongly directional the water displacement is within the tissue and mean diffusivity (MD), which reflects the average displacement distance independent of orientation. The normal white matter will restrict diffusion parallel to the main fiber direction (leading to higher FA and lower MD), whereas white matter damage will cause diffusivity to be less restricted (with lower FA and higher MD) (**10**).

Over the past ten years, interest in the diffusion anomalies of the spinal cord has been growing, with exciting results.

Studies have repeatedly shown altered water diffusion in the CST of ALS patients, with decreased FA and increased MD (**10**). The DTI parameters were well correlated with scores for disease severity (ALSFRS-R, spinal ALSFRS-R, MRC score and hand grip strength), forced vital capacity and mean finger and foot tapping speed (**16**). The differences in FA between ALS patients and controls tend to be more pronounced as we examine the lower cervical segments, supporting the dying back hypothesis of neurodegeneration (**5**)(**17**).

Baseline measurements were not associated with their changes over time. No correlation was found between the longitudinal changes of cord average MD and FA and the concomitant change in the cord area. Cord MRI changes did not differ between rapidly and non-rapidly progressing patients (**18**).

Tensor metrics are sensitive to a variety of factors. A known problem is that axonal loss and swelling, demyelination, reactive gliosis and the presence of cell debris from degenerated fibers can lead to a ‘‘pseudonormalization’’ of mean diffusivity values, which would reduce fractional anisotropy (**19**). Also, the presence of multiple fiber populations (crossing fibers within a single voxel) confounds the results (**10**).

The spinal findings contrast with DTI studies of the CST at the cerebral level where alterations along the CST correlate with the clinical progression measured through ALSFRS-R decline, leading to the hope that DTI-based metrics can be considered as a possible noninvasive follow-up marker for for the progression of the disease (**20**).

## Spinal cord morphometry

Progressive loss of fibers in the lateral columns and motor neurons in the anterior horns has led to the idea that progressive spinal atrophy could be a feature of ALS and this was proven through MRI imaging. Agosta et al. found that cross-sectional area decreases over time on longitudinal spinal cord imaging in ALS and this fact has been replicated in multiple research papers (**13**)(**18**).

Although not all studies agree, the area of the spinal cord seems to be the most sensitive MRI parameter to detect longitudinal disease progression and and correlates significantly with clinical deterioration (**18**)(**21**).

In a 2017 study, spinal cord measurements were predictive of survival, with cervical spine atrophy at C3–C4 and C5–C6 levels correlating with shorter survival. The MRI parameters were more predictive from this point of view compared to the clinical variables (**6**). New data from 2018 suggests that early atrophy of the cervical spinal cord may predict the progression of respiratory distress and could be considered a primary endpoint in neuroprotection studies (**22**).

The spinal cord cross-sectional area seems to be a more reliable marker for longitudinal neurodegeneration than DTI metrics (**6**).

## Current limitations and future directions

Imaging the spinal cord is challenging itself because of the spatial inhomogeneity of the magnetic field in this region, the small size of the spinal cord, the physiological respiratory and cardiac movements (**6**)(**17**). New techniques such as inhomogeneous magnetization transfer (ihMTR) are correlated with ECG and manage to cancel the artifacts generated by the heart movements.

Another limitation is the supine position required during MRI. ALS patients with swallowing or respiratory difficulties are often unable to lie down motionless for a long time in the scanner. This is a known universal selection bias in ALS which constantly prevents the inclusion or follow-up of these patients (**6**)(**18**). This becomes even more significant when applied to complex research protocols in which periods are prolonged (**16**). There are constant efforts in this direction to shorten the duration of the exam without compromising quality, especially by developing multi-slice and multi-angle capabilities for both DTI and ihMTR, providing multiple pure cross-sectional spine slices within a single acquisition (**16**).

Afterwards, there are the classical difficulties in gathering large cohorts in a disease with a medium incidence of 2-3/100.000 and high study dropout rates which are usually 25-30% but can reach 75% (**18**)(**23**). This is usually due to disability progression, severely affected patients becoming unable to come for scheduled visits or to repeat MRI scans, or death (**21**).

With *the advent of 3T MRI,* anatomical spatial resolution and white matter/grey matter contrast are continuing to improve and this is also expected from spinal metrics. Current limitations are the examination duration, the limits of energy transfer to the patient and an increased propensity for artifacts. In 2017, a French team using a 3T MRI observed directly for the first time in ALS the changes in the cervical grey matter, mostly through atrophy measurements, but also through axial diffusivity, FA and ihMTR variations (**16**).

*Inhomogeneous magnetization transfer (ihMTR)* is a new technique, with protocols still being refined. It has a high specificity for myelin due to its unique architecture, consisting of multiple sheaths of lipid bilayers (**24**). The ihMTR technique is magnetic field independent and has been successfully applied for 1.5T and 3T (**16**)(**24**).

ihMTR decreases were found to be systematically more important and more statistically significant than DTI and conventional magnetization transfer metric variations suggesting a marked sensitivity of the technique to microstructural changes of the tissue. This technique will therefore be of great interest when performing longitudinal follow-up on patients with ALS to monitor spatial and temporal tissue demyelination (**16**).

*Progressive iron accumulation* in the spinal cord has been documented on autopsy in ALS patients and mouse models of ALS and iron accumulation has been recently observed in vivo using 7T MRI (**22**). Iron deposits may be a promising biomarker early in the disease and although out of reach yet, they are considered worthy of future study.

*Functional MRI (fMRI)* of the brain has been used extensively in ALS research. To date, no spinal fMRI study has been published in these patients, presumably due to the technical challenges combined with the challenges of gathering and following up an ALS cohort. However, as the technology becomes more widely available, spinal cord fMRI should show promise as a marker in ALS (**13**).

Just as importantly, the current dominant concept is that the best approach to spinal MRI in ALS, at least for the time being, are multiparametric studies, either linking clinical data with different MRI parameters, or various MRI markers between themselves. The results so far are encouraging, at least two studies showing the ability of multiparametric analysis of predicting survival (**6**)(**25**).

Also, there is the relatively recent idea that MRI parameters referring to white matter and grey matter degeneration should be considered together since they mirror the complexity of pathological changes happening in vivo (**6**).

## Discussion

MRI is a constantly expanding field, with new techniques appearing almost every year. It is a unique non-invasive tool that has garnered tremendous potential in evaluating the in vivo evolution of neurodegenerative diseases, both structurally and functionally.

MRI been shown to be useful in the assessment of both white and grey matter in ALS patients, showing damage to the CST, non-motor areas and the spinal cord (**21**). It is hoped that spinal cord MRI could be an effective biomarker and would be widely disposable and noninvasive (**6**). It is likely that the early detection of ALS patients and their inclusion in clinical trials along with multidisciplinary care would lead to better disease outcomes (**4**).

DTI has demonstrated pervasive anomalies of FA and MD in the CST of ALS patients, which tend to correlate with disease severity and may have a particular niche in the detection of occult UMN involvement, thereby expanding the trial inclusion pool (**10**). As of now, they are yet unsuitable for longitudinal follow-up and remain useful only in trials. The normal range of DTI metrics values is wide and in the current phase, we cannot assign a patient as normal or abnormal, outside of transversal or longitudinal comparison.

Spinal cord atrophy (usually determined through area measurements), reflecting both the loss of LMNs and depopulation of the CST, seems to be a more reliable marker for longitudinal studies than DTI (**6**). It also correlates well with disease progression and could be considered a primary endpoint in therapeutic studies (**22**).

Multiparametric approaches are likely to improve the sensitivity of spinal MRI methods and generate valuable predictive data, at least for the near future.

With multiple research avenues appearing on the horizon, the increasing usage of improved 3T machines, functional MRI and new protocols such as ihMTR, the future does not look as bleak as it used to.

## Conflict of Interest

The authors declare that there is no conflict of interest.
